# Correlation of The Etiology of Infertility with Life Satisfaction and Mood Disorders in Couples who Undergo Assisted Reproductive Technologies

**DOI:** 10.22074/ijfs.2017.4658

**Published:** 2017-09-03

**Authors:** Behnaz Navid, Maryam Mohammadi, Samira Vesali, Marzieh Mohajeri, Reza Omani Samani

**Affiliations:** 1 Department of Epidemiology and Reproductive Health, Reproductive Epidemiology Research Center, Royan Institute for Reproductive Biomedicine, ACECR, Tehran, Iran; 2 Department of Psychology, Faculty of Educational Sciences and Psychology, University of Al Zahra, Tehran, Iran

**Keywords:** Anxiety, Depression, Reproduction, Infertility

## Abstract

**Background:**

This study compared common psychological symptoms and life satisfaction in husbands and wives according to infertility diagnosis.

**Materials and Methods:**

We conducted this cross-sectional study on 248 infertile couples between November 1, 2014 and February 28, 2015 at Royan Institute, Tehran, Iran. Participants answered three questionnaires. First, they completed a demographic questionnaire followed by the Hospital Anxiety and Depression Scale (HADS, 14-item self-report instrument) composed of two sub-scales: anxiety (HADS-A) and depression (HADS-D). Participants also completed the Satisfaction with Life Scale (SLWS) comprised of 5 items. Both our questionnaires were validated for the Iranian population.

**Results:**

In couples with male factor infertility, wives had a significantly higher mean score for anxiety compared to their husbands (P<0.001). When the cause of infertility was female factor, the wives appeared significantly more anxious (P<0.001) and depressed (P=0.004) than their husbands. Male patients, those with unknown and female factors, expressed greater satisfaction with life compared to other male patients (P=0.022). Significantly greater depression existed among the couples in which the wives’ educational levels was above their husbands (P=0.045).

**Conclusion:**

Our findings showed that when the infertility etiology was male factor, female factors or unexplained, wives showed significantly higher anxiety than their husbands. In couples diagnosed with female factor infertility, wives showed significantly more depression than their husbands.

## Introduction

Childbearing is an important, valuable issue in marital relationships, especially in traditional societies because it stabilizes the family and increases marital satisfaction (1). After attempts at pregnancy over an extended period of time, the inability to have a child may cause marital problems (2). Most infertile couples report loss of self-esteem, sexual stress, depression, anxiety, guilt, frustration, emotional distress, tension in their marital status, and reduced life satisfaction (3). Most commonly reported forms of infertility related mood disorders are anxiety and depression. These disorders are influenced by a number of factors such as gender, cause of infertility, uncertain treatment duration, financial stress, and pressure from others (4). Several studies have reported the psychosocial impacts of an infertility diagnosis on men and women (1). Infertile couples showed higher rates of psychological symptoms when they had female factor and unexplained causes for infertility. Women have been shown to experience more stress and depression, along with less marital satisfaction compared to their partners. This finding is most probably due to increased involvement in therapeutic procedures, which affects women more (5). Men diagnosed with male factor infertility have more negative emotional responses than other infertile men. They have expressed feelings of stigma and loss of self-esteem (6). It is reported that individuals diagnosed with male factor infertility have greater stress and sexual concern than those with either idiopathic or female factors (7).

Life satisfaction is defined as an assessment of feelings and attitudes about a person’s life at a particular time that ranges from negative to positive and it is a cognitive, judgmental process based on a comparison of individual circumstances to an appropriate standard (8). Research has found that infertile women expressed less satisfaction with their lives compared to fertile women (9). Some studies have focused on marital adjustment, sexual functioning and marital satisfaction in infertile patients (10, 11). Relatively little is known about the influencing factors on life satisfaction in women and men who undergo assisted reproductive technology (ART). We have conducted this study to investigate anxiety, depression, and life satisfaction in Iranian infertile couples who experienced ART.

## Materials and Methods

We conducted this cross-sectional study at Royan Institute, a referral infertility clinic in Tehran, Iran. The sample size was 280 couples from which we gathered 248 completed questionnaires from these couples, for a response rate of 88.5%. Inclusion criteria were: 18 years of age or older, first time undergoing ART, no history for treatment of psychological disorders or chronic diseases, and ability to read and write in Persian. The Ethical Committee of Royan Institute approved this study. Participants received clear explanations about the study aims and data confidentiality. Eligible individuals were assured that acceptance or refusal to participate in the research was voluntarily had no influence on their treatment procedures. Voluntary completion of the questionnaires was considered as consent.

Participants completed three questionnaires. The demographic and fertility information questionnaire included age (years), sex, educational levels (illiterate/under diploma/diploma/academic), duration of infertility (years), and cause of infertility (male factor/female factor/both/unknown). The second questionnaire, the Hospital Anxiety and Depression Scale (HADS) was published in 1983 by Zigmond and Snaith (12). This is a 14 item self-report instrument composed of two subscales: anxiety (HADS-A) and depression (HADS-D). Both consist of 7 items scored on a 4-point Likert scale (0 to 3). In both subscales, 8 items require reverse scoring; then, the anxiety part (HADS-A) and depression (HADS-D) totals can be summed. Both HADS-A and HADS-D scores range from 0 to 21 where higher scores indicate a higher level of anxiety and depression.

The Persian version of HADS has been validated with a Cronbach’s alpha coefficient of 0.78 for HADS-A and 0.86 for HADS-D (13). In the current study, we have determined Cronbach’s alpha scores to be 0.88 for HADS-A and 0.77 for HADS-D. The Satisfaction with Life Scale (SWLS) was published in 1985 by Diener et al. (8). This scale measures global life satisfaction and subjective well-being, and does not tap related constructs such as positive affect or loneliness. It includes 5 items scored on a 7-point Likert scale, that ranges from 1 to 7. The Iranian version of SWLS as determined by Maroufizadeh et al. (14), has a Cronbach alpha coefficient of 0.887. The current study Cronbach alpha coefficient was determined to be 0.85.

### Statistical analysis

We used SPSS version 16.0 (SPSS Inc, Chicago, IL, USA) for statistical analyses. Continuous variables were expressed as mean ± SD and categorical variables as number (%). The relationship between individual independent variables (demographic and fertility characteristics), and dependent variables (anxiety, depression, and SWLS) were assessed by the Pearson correlation coefficient, paired t test (between wives and husbands), independent samples t test (between men and women), and one-way analysis of variance (ANOVA) between causes of infertility followed by the Duncan post hoc test. P<0.05 was considered statistically significant. After paired and pulled analyses, as the mood of husbands and wives influenced each other, we defined a new variable which considered a single, mean score for each couple. We called this analysis “couple analysis” (CA).

## Results

Participants had a mean age of 33.25 ± 5.70 years for men and 29.15 ± 5.28 years for women. We observed no significant difference in depression between men (5.50 ± 3.63) and women (6.65 ± 4.09). Women (8.96 ± 5.00) had significantly more anxiety compared to men (6.04 ± 3.86, P<0.001). No significant difference existed between men (24.46 ± 7.07) and women (23.64 ± 7.06) in terms of SWLS. CA showed that SWLS had a value of 24.05 ± 6.07, anxiety of 7.50 ± 3.58, and depression was 6.07 ± 3.22 for couples. Only 97 (39.1%) men and 95 (38.5%) women had academic educations. In 25% of our couples, wives had a higher educational level compared to their husbands; 24% of husbands had a higher educational level compared to their wives; and the remaining 51% had the same educational level. No significant difference existed between the different educational levels among husbands and wives in SWLS and anxiety (P>0.05). However, there was significantly greater depression among couples in which the wives had a higher educational level than their husbands (P=0.045) or their educational levels were same (P=0.018) compared to those in which the husbands had higher educational levels. According to the cause of infertility, 98 (39.5%) couples had male factor infertility, 76 (30.6%) had female factor, 23 (9.3%) had both, and 51 (20.6%) were unknown. The mean duration of infertility was 4.82 ± 3.50 years in couples. Table 1 lists the participants’ demographic and fertility characteristics.

**Table 1:** Demographic and fertility characteristics of participants (n=248 couples)


		Men Mean ± SD or n (%)	Women Mean ± SD or n (%)	P value

Age (Y)		33.25 ± 5.70	29.15 ± 5.28	<0.001
Education				0.899
	Illiterate	2 (0.8)	2 (0.8)	
	Under diploma	65 (26.2)	59 (23.9)	
	Diploma	84 (33.9)	91 (36.8)	
	Academic	97 (39.1)	95 (38.5)	
Location				-
	City	224 (90.3)		
	Village	24 (9.7)		
Cause of infertility				-
	Male factor	98 (39.5)		
	Female factor	76 (30.6)		
	Both	23 (9.3)		
	Unknown	51 (20.6)		
Duration of infertility (Y)		4.82 ± 3.50		-


### Gender differences in anxiety, depression, and the Satisfaction with Life Scale in male factor group

In couples with male factor infertility, wives had a significantly greater mean score for anxiety compared to their husbands (P<0.001). No significant difference existed between husbands and wives for the HADS-D (P=0.960) and SWLS (P=0.594, Table 2).

### Gender differences in anxiety, depression, and the Satisfaction with Life Scale in the female factor group

In couples with female factor infertility, the wives had significantly higher mean scores for anxiety (P<0.001) and depression (P=0.004) compared to their husbands, but no significant difference was seen in SWLS (P=0.094, Table 2).

### Gender differences in anxiety, depression, and the Satisfaction with Life Scale in the both factor group

In this group, no significant differences existed in anxiety, depression, and SWLS between wives and husbands (Table 2).

### Gender differences in anxiety, depression and the Satisfaction with Life Scale in the unknown factor group

In couples with unexplained infertility, there was no significant difference in depression and SWLS between husbands and wives. Wives had a significantly higher mean score for anxiety than their husbands (P=0.041, Table 2).

### The influence of infertility etiology on anxiety, depression and the Satisfaction with Life Scale

The Kruskal-Wallis test was used separately for males and females for anxiety, depression, and SWLS to identify differences according to the diagnosis of infertility. No significant difference existed among the male patients in anxiety and depression between different causes of infertility (Table 2). In contrast, males with unknown factor fertility had significantly greater SWLS compared to other males (P=0.022). No significant differences were apparent among females in HADS-A, HADS-D, and SWLS between the different etiology groups of infertility (P=0.729, P=0.397, P=0.122 respectively). In CA, we observed no significant differences between couples with different infertility etiologies in SWLS, anxiety, and depression.

### Association between the two questionnaires

Anxiety had a positive, significant association with depression (P<0.001). A significant, negative correlation existed between anxiety and depression with SWLS (P<0.001). These correlations were the same when we analyzed them separately in women and men (Table 3).

**Table 2:** Anxiety, depression, and the Satisfaction with Life Scale (SWLS) in couples and groups


		Male(Mean ± SD)	Female (Mean ± SD)	Both(Mean ± SD)	Unknown(Mean ± SD)	P value^*^

Anxiety						
	Male	6.15 ± 4.11	5.91 ± 3.96	6.13 ± 4.38	6.41 ± 3.74	0.834
Female	8.36 ± 4.60	9.09 ± 5.11	8.35 ± 4.07	8.04 ± 4.16	0.729
P value^**^	<0.001	<0.001	0.071	0.041	
Depression						
	Male	5.94 ± 3.75	5.41 ± 4.28	6.04 ± 4.29	5.24 ± 3.25	0.603
Female	6.29 ± 4.28	6.91 ± 4.12	6.52 ± 3.96	5.65 ± 3.25	0.397
P value^**^	0.690	0.009	0.671	0.508	
SWLS						
	Male	24.40 ± 6.73	25.51 ± 6.28	21.35 ± 7.39	26.31 ± 6.31	0.022
Female	24.12 ± 7.05	23.89 ± 6.62	21.48 ± 7.25	25.63 ± 5.91	0.122
P value^**^	0.594	0.094	0.807	0.578	


*; Test for several independent groups and **; Paired test

**Table 3:** Correlation between anxiety, depression, and the Satisfaction with Life Scale (SWLS)


	Anxiety		Depression		SWLS	
	R	P value	R	P value	R	P value

**Anxiety**	1	-	0.536^*^	<0.001	-0.348^*^	<0.001
**Depression**	0.536^*^	<0.001	1	-	-0.431^*^	<0.001
**SWLS**	-0.348^*^	<0.001	-0.431^*^	<0.001	1	-


R; Pearson correlation and *; P<0.05.

## Discussion

To the best of our knowledge this was the first study on life satisfaction in both husbands and wives (couples) who underwent ART. It appeared that when men have superiority in the family, the family had a lower mean depression score. Our results showed that when the husbands’ educational levels were higher compared to their wives, the couples had a significantly lower mean depression score.

Most studies investigated psychological moods in infertile patients and showed that infertile women experienced higher psychological problems especially for anxiety, depression, and stress (15-17). Our results showed no difference between males and females for depression, also a meta-analysis conducted in Iran showed same results about difference between males and females in depression (18). However, in other studies, infertile women reported more depression than men (19, 20). We found significantly more anxiety in females compared to males which supported results from previous studies (3, 19). An Iranian study showed a greater prevalence of anxiety in infertile women that increased with a history of treatment failure. This finding agreed with the current study results (21). We observed no significant difference between males and females in SLWS.

In 2001, Wischmann et al. (22) reported more satisfaction with life in women compared to men which contradicted to findings of the current study. In this study used a questionnaire on life satisfaction which covered more social factors that included vocational life, financial situation, leisure, marriage, self-esteem, sexuality, and living situation along with health but we have used the SWLS introduced by Diener et al. (8). This questionnaire consists of two major components: i. The emotional or affective component and ii. The judgmental or cognitive component. The difference between our study and Wischmann et al. (22) was possibly due to the different type of the questionnaires and cultures.

We could not find any paper on the relationship between etiology of infertility and satisfaction with life, but we found papers on a similar factor-marital satisfaction. In a Chinese study, wives with both male and female factors expressed less marital satisfaction than their husbands (1). Another study in Iran showed that women with female factor infertility had less marital satisfaction than their infertile counterparts (11). Although our study did not evaluate marital satisfaction, we could not find any significant difference in satisfaction with life between the different infertility etiologies among females.

We found that when the infertility etiology was male factor, female factors or unexplained, wives also showed strongly significantly higher anxiety than their husbands. The reason was thought to be caused by the idea that conception and childbirth have been considered women’s responsibility, especially in traditional societies and some developed countries (15). Ramezanzadeh et al. (23) have explained that in Islamic and Middle Eastern countries, such as Iran, childbearing is very important and leads to family stabilization and increased marital satisfaction. They also reported that in these countries negative attitudes toward infertility exist such as stigmatization, marital instability, divorce, and abuse for infertile women. Therefore, involuntary childlessness may be associated with enhanced psychological problems. In our study, in couples who suffered from female factor infertility, wives showed significantly more depression than their husbands.

Wischmann et al. (22) found that anxiety and depression in couples with unexplained factor infertility were higher than couples with other factors, and in women more than men. A study reported that less anxiety and depression in females whose male partners were infertile (24), but we did not observe these results in the current study. Ogawa et al. (2) reported that females with knowledge of their male partner’s infertility had lower anxiety scores on the HADS test than infertile women who did not have such knowledge. Our study could not confirm this finding

The current study had several limitations. First, we have relied on infertile couples who presented to a single center. However this is a referral clinic for infertility treatment that treats patients from all of Iran. Second, the cross-sectional nature of the study only reveals a correlation, but no conclusion on causality. Additional longitudinal studies are required to explore the direction of causality and to determine how the study variables may change over the course of the infertility treatment.

## Conclusion

Infertile women with female and male factor showed higher anxiety and depression compared to their husbands. Couples with unknown factor showed no significant differences in depression and SWLS between husbands and wives. SWLS was higher in men with unknown factor compared to other males. Women with female factor expressed more anxiety and depression than women with male factor infertility, but SWLS did not differ among these women. It has been suggested to add psychological counseling before and during treatment cycles.
